# Olfactory inputs to appetite neurons in the hypothalamus

**DOI:** 10.1073/pnas.2524926123

**Published:** 2026-01-27

**Authors:** Donghui Kuang, Naresh K. Hanchate, Chia-Ying Lee, Ashley Heck, Xiaolan Ye, Michidsaran Erdenebileg, Charu Mehta, Md Mehedi Hassan, Manu Setty, Linda B. Buck

**Affiliations:** ^a^Basic Sciences Division, Fred Hutchinson Cancer Center, Seattle, WA 98109; ^b^Public Health Sciences Division, Fred Hutchinson Cancer Center, Seattle, WA 98109; ^c^The Brotman Baty Institute for Precision Medicine, Seattle, WA 98195

**Keywords:** olfactory, appetite, circuits

## Abstract

The sense of smell has potent effects on appetite, but the underlying neural pathways remain undefined. Here, we investigated how olfactory signals reach two subsets of appetite-linked (“appetite”) neurons in the hypothalamic arcuate nucleus: Agouti-related peptide (AgRP) neurons, which stimulate appetite, and POMC (pro-opiomelanocortin) neurons, which suppress it. Using polysynaptic viral tracing, we show that AgRP and POMC neurons receive indirect input from partially overlapping but distinct areas of the olfactory cortex, indicating that they process different sets of olfactory information. We also identify different complements of neurons more directly upstream of AgRP and POMC neurons that could relay olfactory cortical signals to the appetite neurons. Single-cell transcriptomics shows heterogeneous expression of neuromodulator receptors among AgRP neurons, suggesting variations in the signals they receive. Integrated viral tracing and RNA localization further reveals selected brain areas where upstream neurons express cognate receptor ligands. Together, these findings outline multiple pathways by which distinct olfactory and modulatory signals are differentially routed to neurons that promote versus inhibit appetite.

## Significance

The sense of smell profoundly influences appetite, but the underlying neural circuits are unexplored. Here, we examine circuits upstream of hypothalamic agouti-related peptide (AgRP) neurons, which stimulate appetite, and pro-opiomelanocortin (POMC) neurons, which inhibit it. We find that AgRP and POMC neurons receive indirect input from different combinations of olfactory cortical areas and thus process different sets of olfactory signals. We also identify different complements of neurons more directly upstream of AgRP versus POMC neurons that could relay olfactory signals to them. RNA analyses reveal differential expression of neuromodulator receptors among AgRP neurons and cognate receptor ligands in upstream neurons in specific brain areas. These findings uncover multiple pathways for linking the sense of smell to hypothalamic appetite neurons.

The regulation of appetite and food intake is essential for survival and is conserved across the animal kingdom. In mice and other mammals, the arcuate nucleus of the hypothalamus (ARC) contains two subsets of neurons with opposite roles in appetite regulation: Agouti-related peptide (AgRP) neurons, which promote appetite, and POMC neurons, which inhibit it. AgRP neurons express agouti-related peptide, while POMC neurons express pro-opiomelanocortin (POMC) (1, 2).

AgRP neurons are activated when animals are calorically deficient (3–5). Optogenetic or chemogenetic activation of these neurons stimulates voracious eating and other hunger-related behaviors (6–8). Activated AgRP neurons can also induce lipogenesis, fat mass accumulation, and altered substrate utilization (9). In contrast, POMC neurons are stimulated when energy is available (5), and optogenetic activation of POMC neurons inhibits feeding even in fasted animals (10).

The smell and sight of food dramatically alter AgRP and POMC neuron activity (11–13). Within seconds before food is consumed, the activity of AgRP neurons is inhibited while POMC neurons are stimulated. These effects appear to arise from learned associations between sensory food cues and the caloric or nutritional value of the food (11, 14).

How is the smell of food conveyed to AgRP and POMC neurons to elicit these effects on appetite? In the olfactory system, odor signals travel from the nasal olfactory epithelium through the olfactory bulb to the olfactory cortex (OC), which transmits information to multiple other brain areas (15, 16). The OC comprises a number of anatomically distinct areas whose respective functions are poorly understood (17–19). Whether one or more of these areas is involved in appetite is unknown.

Previous studies used monosynaptic rabies virus to map the locations of brain neurons presynaptic to AgRP or POMC neurons (20, 21). Those studies showed neurons presynaptic to AgRP or POMC neurons in multiple brain areas, but not the OC. Those results suggested that odor signals might be conveyed from the OC to AgRP and POMC neurons via indirect routes that cannot be detected using a monosynaptic rabies virus.

To investigate this idea, we infected AgRP or POMC neurons with PRVB177, a conditional Pseudorabies virus that travels retrogradely across multiple synapses after infecting neurons expressing Cre recombinase (22). These experiments revealed infected neurons likely to be two synapses upstream of AgRP and POMC neurons in the OC, confirming that both receive odor signals indirectly from the OC. However, infected neurons were seen in some OC areas but not others, suggesting that only some OC areas provide signals to the appetite neurons. In addition, OC areas upstream of AgRP and POMC neurons overlapped only partially, further suggesting that AgRP and POMC neurons receive different sets of olfactory information.

Neurons more directly upstream of AgRP or POMC neurons were seen in multiple brain areas. These areas provide potential routes by which information could be relayed from the OC to the appetite neurons to affect their activity and thus modulate appetite.

Neurons upstream of AgRP and POMC neurons were also present in two amygdala areas that receive signals from the vomeronasal organ (VNO), an accessory olfactory structure that detects social cues (15, 16). This finding raises the possibility that social cues could influence appetite.

To investigate signaling mechanisms that could relay olfactory information to AgRP neurons, we employed single-cell RNA sequencing to identify AgRP neuron receptors for neuromodulators. We then used viral tracing combined with RNA in situ hybridization to locate upstream neurons expressing cognate neuromodulators. These experiments uncovered numerous neuromodulator receptors differentially expressed by AgRP neurons. They also revealed that cognate ligands of those receptors can be selectively expressed by upstream neurons in specific brain areas.

Together, these studies uncover areas of the OC that convey odor signals to AgRP and POMC neurons and nonolfactory brain areas with the potential to relay those signals from the OC to AgRP and POMC neurons. These studies also unravel signaling mechanisms that can modulate AgRP neurons. They show that single AgRP neurons differentially express diverse receptors for neuromodulators and that ligands of those receptors are selectively expressed in neurons upstream of AgRP neurons in specific brain areas. These findings provide a foundation for molecular-genetic studies to further explore the transmission of olfactory information to AgRP and POMC appetite neurons and signaling circuits and mechanisms underlying the modulation of appetite.

## Results

### Appetite Neurons Receive Indirect Input from the OC.

**Fig. 1.**
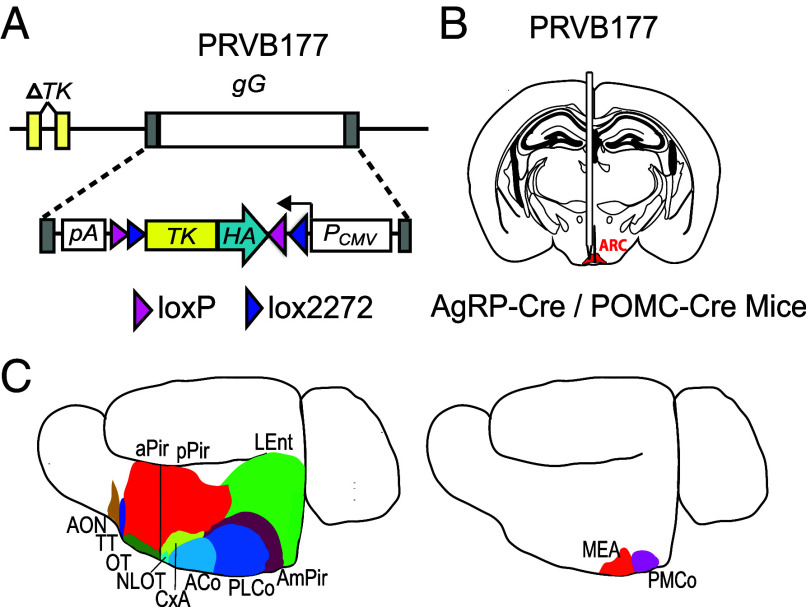
Strategy to assess input to ARC AgRP and POMC neurons from higher olfactory areas. (*A*) Cre recombinase-driven irreversible expression of TK-HA from PRVB177 allows viral replication and synaptic spread. *gG*, *gG* locus; *PCMV*, cytomegalovirus promoter; *pA*, polyadenylation signal. (*B*) Injection of PRVB177 into the ARC (indicated in red) of AgRP-Cre or POMC-Cre mice. (*C*) Immunostaining for PRV (HA) in brain sections to identify infected neurons within the OC (*Left*) or VA (*Right*). Adapted from Kondoh et al. (22).

To investigate whether AgRP and POMC neurons receive input from higher olfactory areas, we utilized PRVB177, a Cre-dependent Pseudorabies virus (Fig. 1). PRVB177 has an irreversible Cre recombinase-dependent hemagglutinin tagged thymidine kinase (HA-TK), which allows it to propagate and travel retrogradely across multiple synapses (22).

PRVB177 was injected into the arcuate nucleus (ARC) of mice expressing Cre in AgRP or POMC neurons (AgRP-Cre or POMC-Cre mice) (Fig. 1). To examine the locations of upstream PRV-infected neurons, brain sections were then immunostained for HA. This method was previously used in CRH-Cre mice to examine neurons upstream of hypothalamic neurons expressing CRH (corticotropin releasing hormone) (22).

In CRH-Cre mice, areas with PRVB177+ neurons on d3pi were largely the same as those labeled with a dual virus, “monosynaptic PRV” system (22), suggesting that PRVB177+ upstream neurons on d3pi in the present studies are likely to be presynaptic to the appetite neurons.

**Fig. 2.**
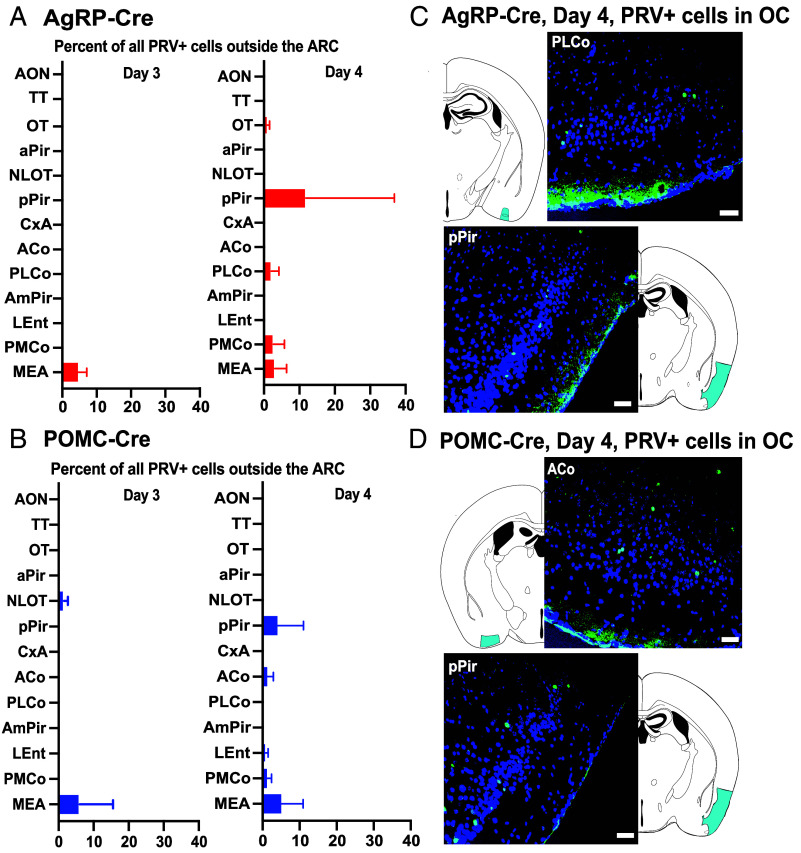
AgRP and POMC neurons receive input from higher olfactory areas. Mean percentages of PRV+ neurons outside ARC in areas of the OC or VA on day 3 or 4 after infection of AgRP (*A*) or POMC (*B*) neurons with PRVB177. Day 3: AgRP-Cre (*n* = 16); POMC-Cre (*n* = 4). Day 4: AgRP-Cre (*n* = 6); POMC-*Cre* (*n* = 4). Error bars indicate SEM. See *Materials and Methods* for full names of abbreviated brain areas. Photographs and diagrams of OC sections with neurons immunostained for PRV on day 4 postinjection of AgRP-Cre (*C*) or POMC-Cre (*D*) mice. PRV+(HA+), green; DAPI counterstain, blue. (Scale bar, 100 μm.) Corresponding areas on diagrams are indicated in cyan.

**Fig. 3.**
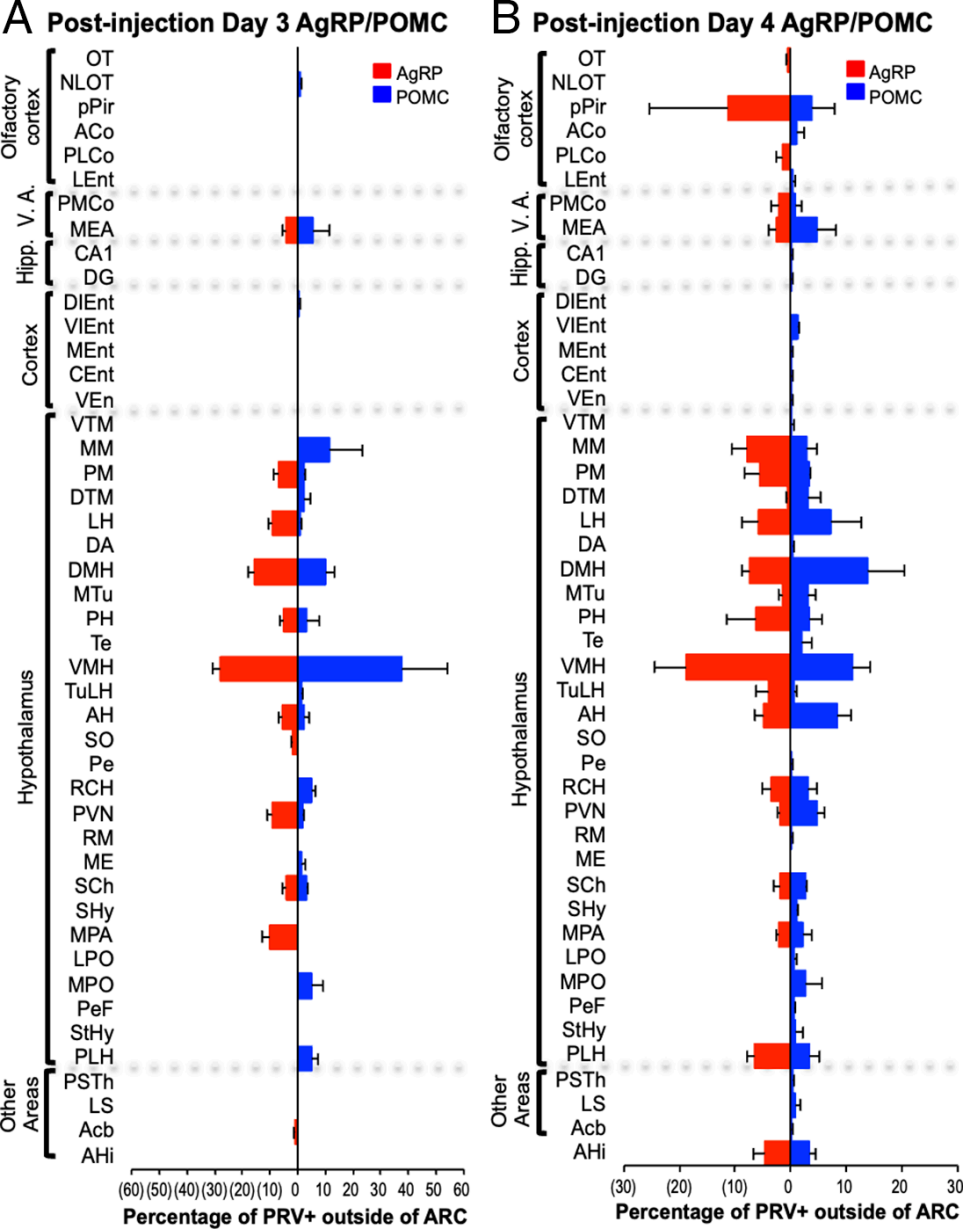
Brain areas with PRV+ neurons after infection of AgRP or POMC neurons. Bar graphs show the percentage of non-ARC PRV+ neurons in individual brain areas on days 3 (*A*) and 4 (*B*) postinfection of ARC AgRP (red) or POMC (blue) neurons. Sample sizes for days 3 and 4 postinjection as in Fig. 2. Error bars indicate SEM. V.A., vomeronasal amygdala; Hipp., hippocampus. See *Materials and Methods* for full names of abbreviated brain areas.

The OC receives odor signals generated in the nose via a relay in the olfactory bulb. The OC comprises multiple anatomically distinct areas whose respective functions are poorly understood (17–19) (Fig. 1). On d3pi, PRV+ neurons were observed in POMC-Cre mice in one OC area, the nucleus of the lateral olfactory tract (NLOT). None were detected in the OC in AgRP-Cre mice (Figs. 2 and 3 and *SI Appendix*, Figs. S1 and S4).

In contrast, on d4pi, PRV+ neurons were seen in the OC of both AgRP-Cre and POMC-Cre mice (Figs. 2 and 3 and *SI Appendix*, Fig. S1). These neurons are likely to be two synapses upstream of the appetite neurons (although >2 synapses upstream cannot be excluded) and thus provide indirect input to them. However, some OC areas contained PRV+ neurons, whereas others did not. Of 11 different OC areas, five contained PRV+ neurons upstream of AgRP or POMC neurons, suggesting that only some OC areas provide signals to the appetite neurons.

In addition, the OC areas with PRV+ neurons differed in AgRP-Cre versus POMC-Cre mice. In AgRP-Cre mice, PRV-infected neurons were identified in three OC areas: the olfactory tubercle (OT), posterior lateral cortical amygdala (PLCo), and posterior piriform cortex (pPir). The percentages of all PRV+ cells outside the ARC in these areas were 0.4 ± 0.3%, 1.5 ± 0.9%, and 11.2 ± 10.2%, respectively. In POMC-Cre mice, PRV+ neurons were seen in a different set of three OC areas: the lateral entorhinal cortex (LEnt), anterior cortical nucleus (ACo), and pPir. The percentages of all PRV+ cells external to the ARC in these three areas were 0.4 ± 0.3%, 1.0 ± 1.0%, and 3.8 ± 3.7%, respectively.

These results indicate that both AgRP and POMC appetite neurons receive information indirectly from the OC. They suggest that one major OC area, the pPir contains neurons two synapses upstream of both AgRP and POMC neurons. They further suggest that there are also neurons two synapses upstream of AgRP neurons in two additional OC areas, the OT and the PLCo and neurons two synapses upstream of POMC neurons in two other OC areas, the ACo and the LEnt. Thus, while one OC area, pPir, provides indirect input to both subsets of appetite neurons, there are four additional OC areas that provide indirect input to only one or the other subset: the OT and PLCo only to AgRP neurons and the ACo and LEnt only to POMC neurons. Thus, AgRP and POMC neurons receive signals from partially overlapping areas of the OC and could thereby receive different sets of olfactory information.

Mice have an accessory olfactory structure in the nasal septum called the VNO, which detects pheromones and other social cues (15, 16, 23). PRV+ neurons were observed upstream of both AgRP and POMC neurons in the “vomeronasal amygdala” (VA), which receives signals derived from the VNO (Figs. 1–3 and *SI Appendix*, Fig. S1). One VA area, the medial amygdala (MEA), contained PRV+ neurons upstream of both AgRP and POMC neurons on d3pi, suggesting that it provides more direct input to the appetite neurons. The other, the posteromedial cortical amygdala (PMCo), showed PRV+ neurons upstream of both types of appetite neurons on d4pi, suggesting indirect inputs to the appetite neurons. These results suggest a means by which social cues detected in the VNO might influence appetite.

Together, these results indicate that the OC provides indirect input to both AgRP and POMC neurons. However, only some areas of the OC do this. Moreover, AgRP and POMC neurons receive input from partially overlapping but distinct OC areas, suggesting that they receive different complements of olfactory information.

### Appetite Neurons Receive More Direct Input from Multiple Brain Areas.

The presence of OC neurons indirectly upstream of AgRP and POMC neurons implied that OC signals are relayed to the appetite neurons by their presynaptic partners in one or more other brain areas. These experiments identified PRV+ neurons more directly upstream of AgRP or POMC neurons in multiple areas that could play this role. These PRV+ neurons were identified on d3pi. In CRH-Cre mice, areas with PRVB177+ neurons on d3pi were largely the same as those labeled with a dual virus, “monosynaptic PRV” system (22), suggesting that PRVB177+ upstream neurons on d3pi in the present studies are likely to be presynaptic to the appetite neurons.

On d3pi of AgRP neurons, PRV+ cells were identified in 11 nonolfactory (non-OC, non-VA) brain areas outside the ARC (Fig. 3 and *SI Appendix*, Figs. S1, S2, and S4). These areas were predominantly in the hypothalamus, a region that regulates endocrine and other basic functions, such as appetite, thirst, and instinctive behaviors (1, 24–30). The highest concentrations of PRV+ cells were observed in the ventromedial hypothalamus (VMH) and dorsal medial hypothalamus (DMH). PRV+ cells were also seen in the nucleus accumbens (Acb), which is linked to reward (31).

On d3pi of POMC neurons, PRV+ neurons were detected in 16 nonolfactory brain areas (Fig. 3 and *SI Appendix*, Figs. S1, S3, and S4). As with AgRP neurons, these areas were primarily located within the hypothalamus, with high concentrations of PRV+ cells in both the VMH and DMH.

Eight nonolfactory brain areas exhibited PRV+ neurons 3 d postinfection of both AgRP and POMC neurons, suggesting that these regions could modulate both neuron types (Fig. 3 and *SI Appendix*, Figs. S1 and S4). It remains to be determined whether the neurons in these areas that project to AgRP and POMC neurons are identical or distinct.

These findings are similar to those from studies employing a monosynaptic rabies virus to trace neurons presynaptic to ARC AgRP or POMC neurons (20, 21). For inputs to AgRP neurons, on d3pi, we found 12 upstream areas, 10 in the hypothalamus, with 1 to 28% of infected cells/area. Using monosynaptic rabies virus, Krashes et al. (20) found 9 upstream areas, all in the hypothalamus, with 1 to 26% of the rabies+ cells/area. And Wang et al. (21) found 34 upstream areas with rabies+ cells, 17 in the hypothalamus, with 0.1 to 13% of the rabies+ cells/area. Differences in the percentages of virus-infected cells/area among these studies are likely due at least in part to different modes of counting infected cells: We immunostained brain sections and counted labeled neurons by eye, Krashes used a fluorophore expressing rabies virus and counted cells without immunostaining, and Wang used immunostaining and automated counting of stained sections.

Nonetheless, several findings were common to the PRV and two rabies virus studies. First, eight infected hypothalamic areas were shared by all three studies. Second, in all three, the highest percentages of infected neurons were seen in specific areas. The DMH contained 15%, 13%, and 26% of infected studies in the PRV, Wang, and Krashes studies, respectively. The VMH contained 28%, 10%, and 1% in those studies, and the PVN contained 9%, 10%, and 18% in the 3 studies.

Similarities were also seen in Inputs to POMC neurons in the PRV and Wang rabies virus studies. For inputs to POMC neurons, we found 18 upstream areas, 15 in the hypothalamus, with 0.4 to 38% of PRV+ cells/area. Wang found 51 rabies+ areas, 17 in the hypothalamus, with 0.1 to 6% of infected neurons/area. Areas with the highest percentages of infected cells were the VMH (38% of PRV+, 5% of rabies+) and the DMH (10% of PRV+ and 6% of rabies+). The MEA contained both PRV+ and rabies+ cells upstream of both AgRP and POMC neurons.

By d4pi, additional nonolfactory brain areas showed PRV+ neurons upstream of AgRP or POMC neurons (Fig. 3 and *SI Appendix*, Fig. S1). As in the OC, the new PRV-infected neurons identified on day 4 are likely to be two synapses upstream of AgRP or POMC neurons and transmit information to the appetite neurons indirectly via relays more directly upstream of the appetite neurons.

These experiments showed twelve brain areas with neurons more directly upstream of AgRP neurons and eighteen with neurons more directly upstream of POMC neurons. The upstream neurons in one or more of those areas could relay signals from the OC to the appetite neurons. Most or all projection neurons in the OC are excitatory glutamatergic neurons. However, they could activate either excitatory or inhibitory neurons presynaptic to AgRP or POMC neurons and thereby either stimulate or suppress the appetite neurons.

As already noted, PRV+ neurons indirectly upstream of AgRP or POMC neurons in the OC suggested that AgRP neurons receive input from OC neurons in the pPir, OT, and PLCo while POMC neurons receive input from those in the pPir, ACo, and LEnt. One question is whether signals from those areas reach the appetite neurons by relays in different areas of the hypothalamus. Early studies using traditional anterograde and retrograde tracers indicated that at least some OC areas, including the piriform cortex, periamygdaloid areas (including PL/MCo), the entorhinal cortex, and ACo, as well as the MEA, project to regions of the hypothalamus, which were not defined as specific areas (32). More recently, the Allen Brain Institute used eGFP-AAVs (adeno-associated viruses) and ultrasensitive tomography to uncover connections among different brain areas, which are available in a searchable online database (33). Using this facility, we obtained evidence that the piriform cortex, PL/MCo, LEnt, and MEA each project to many of the hypothalamic areas likely to be directly upstream of AgRP and/or POMC neurons. However, there was no information available on OT or ACo projections in wild-type animals. Furthermore, since the viral injections in wild-type animals were usually not restricted to a specific area (e.g., piriform cortex), the mapping of specific connections will require validation by additional experiments.

### AgRP Neurons Express Diverse Receptors for Neuromodulators.

**Fig. 4.**
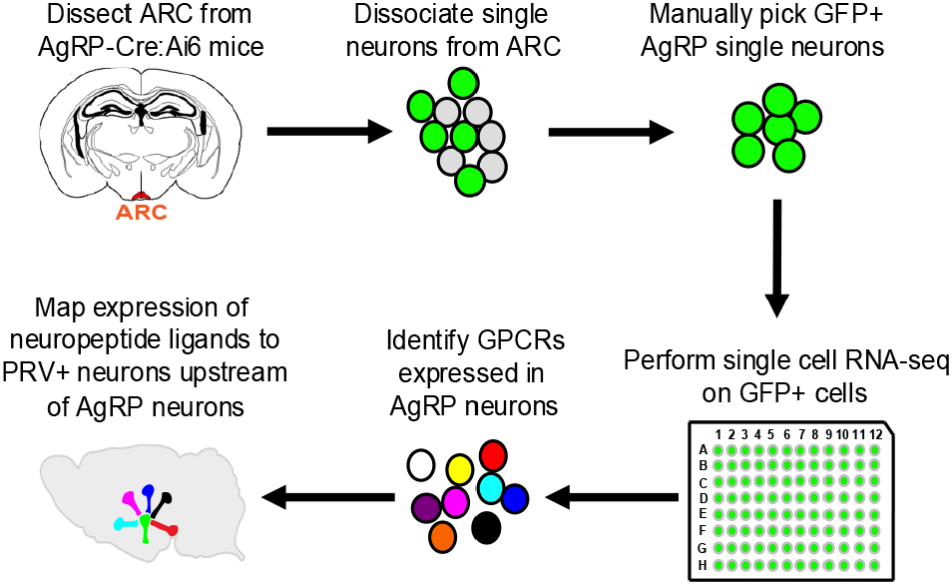
Identification of receptors expressed in AgRP neurons and upstream neurons expressing their ligands. The ARC was dissected from AgRP-Cre:Ai6 mice and dissociated into single cells. Single GFP+ AgRP neurons were manually isolated and subjected to single-cell RNA-seq. Transcriptome data were used to identify receptors expressed in single AgRP neurons and those with known ligands determined. The expression of individual neuropeptide ligands in neurons directly upstream of AgRP neurons was determined by infecting AgRP neurons with PRVB177 and costaining brain sections for PRV and different neuropeptides.

The above experiments traced neurons likely to be directly upstream of AgRP neurons to 12 brain areas, one or more of which could relay information from the OC to AgRP neurons. To explore signaling mechanisms that could relay olfactory information to AgRP neurons, our strategy was to catalog AgRP neuron receptors for neuromodulators and then pinpoint the sources of receptor ligands in upstream neurons (Fig. 4).

To identify AgRP neuron receptors for neuromodulators, single-cell transcriptome analysis was performed on AgRP neurons isolated from the arcuate nucleus. AgRP-IRES-Cre mice (34) were crossed with Ai6 mice (35), which have Cre-dependent expression of eGFP, to generate AgRP-Cre:Ai6 mice. Arcuate nuclei were dissected, dissociated into single cells, and single GFP+ cells were manually transferred to individual tubes. Each cell was subjected to single-cell RNA sequencing (scRNA-seq), employing established protocols to generate and analyze single-cell cDNA libraries (27, 36, 37). On average, 18 cells were sequenced at ~9.5 million reads per cell, with an average of 3,952 genes detected per cell. Sequenced reads were mapped to the mouse genome (UCSC mm10, GENCODE M15). Gene expression was quantified, setting a threshold of 1 FPKM (fragments per kilobase of transcript per million mapped reads) for expression in individual cells.

**Fig. 5.**
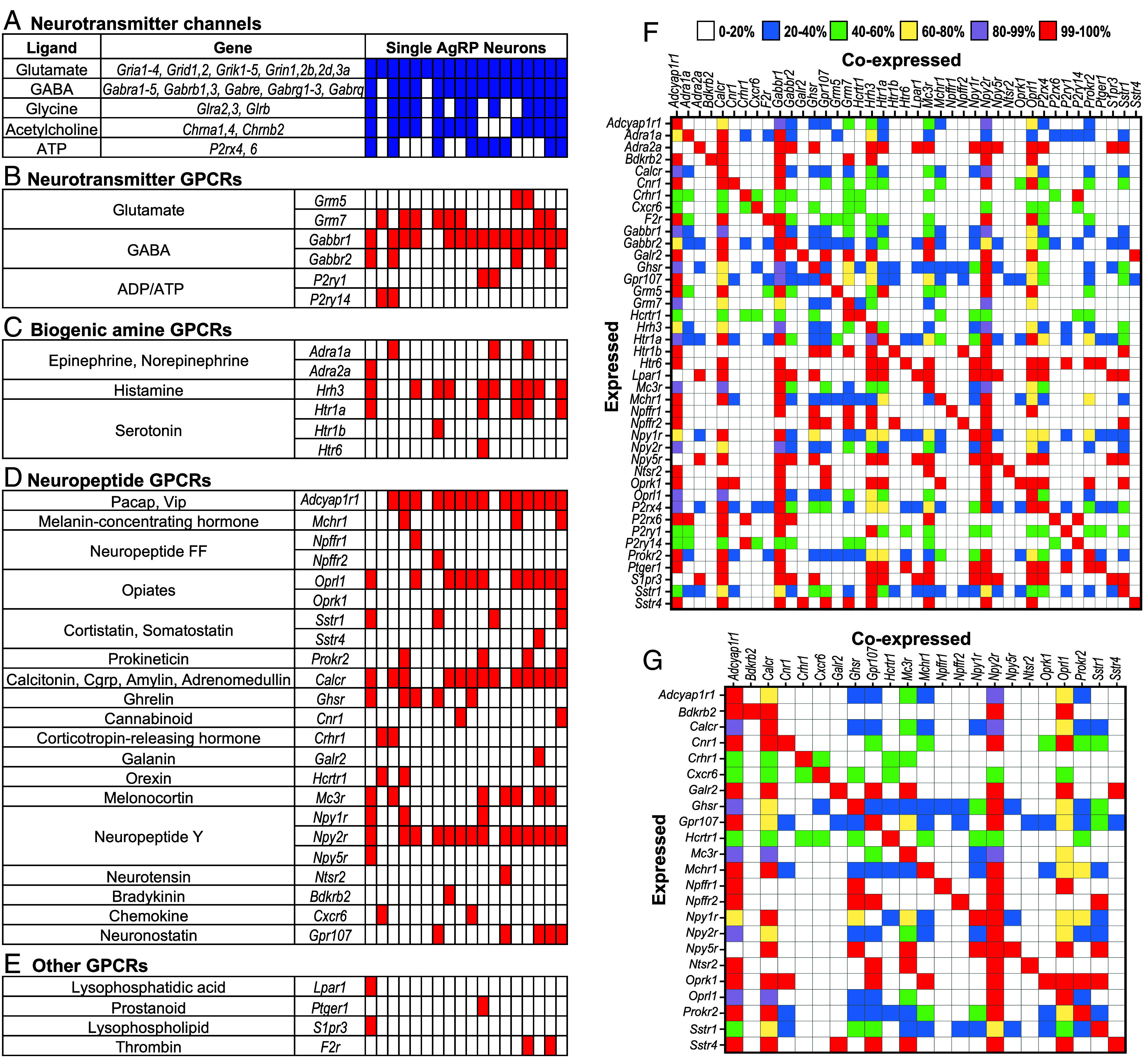
AgRP neurons express multiple receptors for neuromodulators. Transcriptome data from individual AgRP neurons indicate that single AgRP neurons can express genes encoding multiple neurotransmitter receptor channels and GPCRs and that those genes are expressed in different combinations in different neurons. Shown here are neurotransmitter receptor channels and GPCRs with known ligands indicated on the *Left*. Colored boxes indicate expression in individual AgRP neurons. Blue boxes show neurotransmitter receptor channels expressed in AgRP neurons (*A*). GPCRs expressed in AgRP neurons are indicated by red boxes and include GPCRs for neurotransmitters (*B*), biogenic amines (*C*), neuropeptides (*D*), and other ligands (*E*). Coexpression data are shown for all GPCRs (*F*) or neuropeptide GPCRs (*G*). Expressed genes are indicated on the y-axis and coexpressed genes on the x-axis. Box colors indicate percentages of neurons expressing different genes (y-axis) that coexpress genes on the x-axis.

This analysis revealed a diverse array of 39 G protein–coupled receptors (GPCRs) expressed by AgRP neurons (Fig. 5 and *SI Appendix*, Fig. S5) (38). These included six metabotropic receptors for the neurotransmitters glutamate, GABA, or ADP/ATP, six receptors for the biogenic amines (epinephrine/norepinephrine, histamine, or serotonin, 23 receptors for neuropeptides, and four for other signaling molecules.

AgRP neurons also exhibited expression of ionotropic (ligand-gated ion channel) receptors for neurotransmitters (38) (Fig. 5). All expressed ionotropic receptors for glutamate, the major excitatory neurotransmitter in the brain. Many also expressed ionotropic receptors for GABA, the major inhibitory neurotransmitter in the brain. And some also expressed ionotropic receptors for glycine, acetylcholine, and/or ATP.

These findings resemble previous RNA-seq data from grouped AgRP neurons (39). However, the present data go beyond those data by providing information about individual AgRP neurons.

The present data make several important points. First, individual AgRP neurons can respond to multiple different GPCR ligands. On average, each neuron expressed 9 different GPCRs. Most expressed one or more neuropeptide GPCRs, with individual neurons expressing an average of 6 such receptors. Many also expressed one or more GPCRs for biogenic amines, neurotransmitters, or other ligands.

Second, individual AgRP neurons show considerable heterogeneity in the specific combination of GPCRs they express (Fig. 5). Thus, different neurons may respond to different combinations of GPCR ligands. This finding is similar to those we obtained by scRNA-seq of CRH neurons in the paraventricular nucleus (PVN) of the hypothalamus (28).

Third, the prevalence of specific GPCRs varies among neurons. For example, the neuropeptide receptor Adcyap1r1 was found in 78% of neurons while the neuropeptide receptor Galr2 was identified in only 6% (Fig. 5 and *SI Appendix*, Fig. S5). Given that we conducted deep sequencing of a relatively small number of AgRP neurons, the percentage expressing individual receptors could vary due to dropout effects during RNA-seq.

To compare our results with another dataset with a large number of AgRP neurons, we extracted scRNA-seq data on 4,174 AgRP+ neurons from the ARC in the Allen Brain Cell Atlas (40). For this atlas, scRNA-seq was conducted on roughly 4 million mouse brain cells. Transcripts were sequenced at approximately 55-85 thousand reads/cell with a threshold of 2,000 genes/cell.

In the 4,174 AgRP+ neurons, we found 33 of 39 GPCRs identified in our dataset using a threshold of 1 CPM (counts/million) for expressed genes. As in our dataset, the percentage of neurons with different receptors varied. In some cases, but not all, the prevalence was similar in the two datasets. For example, Gabbr1, Adcyapr1, and Npy2r were found in 83%, 78%, and 78% of cells in our dataset and 100%, 86%, and 61% of cells in the Allen dataset. And, at the other extreme, Hcrtr1, Bdkrb2, and Ptger1 were found in 11%, 6%, and 6% of cells in our dataset and 5%, 4%, and 2% of cells in the Allen dataset.

As in our dataset, AgRP neurons in the Allen dataset showed expression of different sets of GPCRs per cell, and the combinations were heterogeneous among neurons (*SI Appendix*, Figs. S6 and S7).

Together, these results suggest that individual AgRP neurons can vary considerably in the combination of GPCRs they express in addition to ionotropic neurotransmitter receptors. The complement of GPCRs they express could enhance or dampen the major excitatory and inhibitory signals they receive via the ionotropic neurotransmitter receptors and thus fine-tune their responses to signals from upstream neurons and, in turn, the signals they send to downstream neurons to modulate appetite.

### Ligands of AgRP Neuron Receptors Define Subsets of Upstream Neurons.

The viral tracing experiments identified neurons more directly upstream of AgRP neurons in 12 brain areas. The single-cell transcriptome experiments revealed AgRP neuron receptors for multiple signaling molecules, including ionotropic neurotransmitter receptors and GPCRs for neurotransmitters, biogenic amines, and neuropeptides. One key question to understanding the neural circuits controlling appetite is whether there are links between the anatomical locations of upstream neurons and the GPCR ligands they use to communicate with AgRP neurons.

To address this question, we employed the Receptor-Assisted Mapping of Upstream Neurons method we previously used to characterize signaling molecules in neurons upstream of CRH neurons (27). We infected AgRP neurons with PRVB177, allowed 3 d for the virus to traverse one synapse, and then costained brain sections for PRV and individual neuropeptides (Fig. 4).

**Fig. 6.**
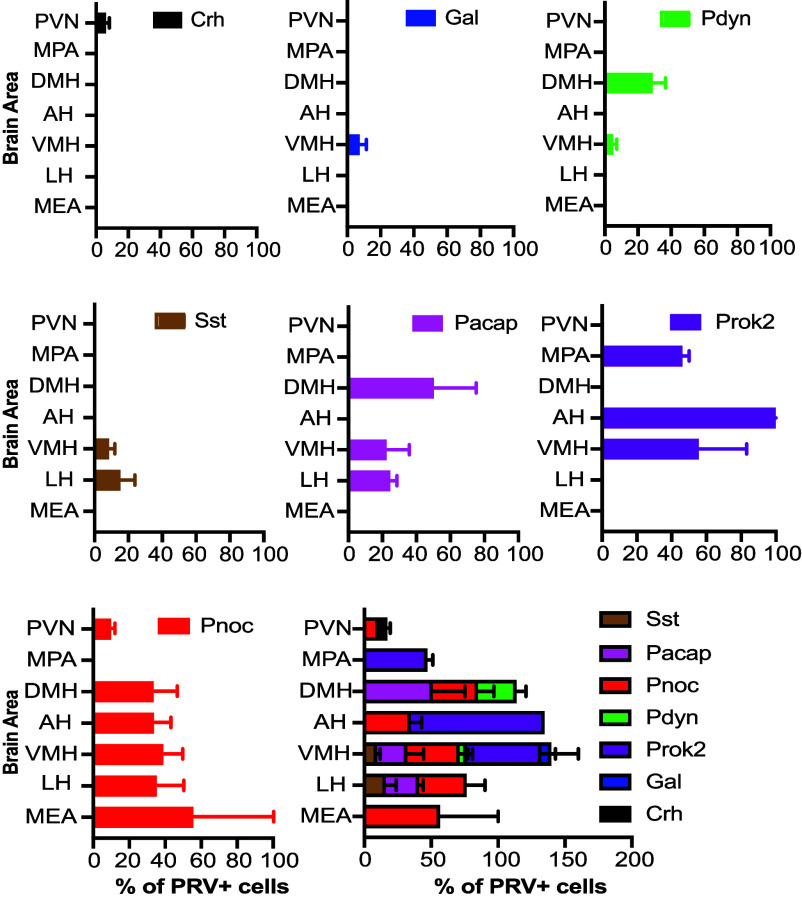
Neuropeptide ligands of AgRP neuron receptors are expressed by upstream neurons in specific brain areas. Graphs show percentages of PRV+ neurons labeled for specific neuropeptides in different areas on day 3 after infection of AgRP neurons with PRVB177 (n = 3-4). Error bars indicate SEM. See *Materials and Methods* for full names of abbreviated brain areas.

We analyzed the expression of seven neuropeptides in PRV+ neurons likely to be directly upstream of AgRP neurons (Fig. 6 and *SI Appendix*, Fig. S8). The distribution of these neuropeptides varied widely. For example, Crh was detected exclusively in PRV+ cells within the PVN, while Pnoc was found in PRV+ cells in six different areas.

The proportion of PRV+ cells expressing specific neuropeptides also varied. For example, only 6.3 ± 1.6% of PRV+ cells in the PVN expressed Crh, and 7.6 ± 3.8% of PRV+ cells in the VMH expressed Gal. At the other extreme, every PRV+ neuron in the anterior hypothalamus (AH) expressed Prok2.

In addition, a given neuropeptide was observed in different percentages of PRV+ neurons in different areas. For instance, Pnoc was present in 10.0 ± 1.7% of PRV+ cells in the PVN compared to 55.9 ± 44.1% in the MEA.

The total percentage of the PRV+ neurons expressing different neuropeptides in DMH, AH, and VMH was over 100%, suggesting that there are upstream neurons expressing more than one of the neuropeptides in these brain areas (Fig. 6). This is reminiscent of “Connect-seq” experiments where we found 62% of neurons upstream of CRH neurons expressing two or more neuropeptides (38).

We observed no correlation between the total number of PRV-infected neurons and the percentages of neurons labeled for specific neuropeptides in different areas, indicating that the variations in neuropeptide expression are not simply due to differences in viral infection rates. In addition, there was no apparent correlation between the number of upstream neurons expressing a particular neuropeptide and the proportion of AgRP neurons expressing the corresponding receptor.

Together, these results show that neuropeptide ligands of AgRP-neuron GPCRs are differentially expressed in upstream neurons in a number of areas, primarily in the hypothalamus. Altogether, seven neuropeptide ligands were seen in seven of the twelve areas likely to be upstream of AgRP neurons. Their distribution within that set of areas varied, but it clearly suggests that the neuropeptide ligands tend to be selectively expressed in upstream neurons in specific brain areas. These results highlight routes of transmission of olfactory and other cues to AgRP neurons and signaling mechanisms employed in those routes to modulate AgRP neurons and their effects on appetite.

## Discussion

The olfactory system has profound effects on appetite and food intake. Here, we sought to gain insight into the mechanisms and neural circuits that underlie these effects. We focused on two subsets of appetite-linked neurons in the hypothalamic arcuate nucleus: AgRP neurons, which stimulate appetite, and POMC neurons, which suppress it.

To examine neural circuits that convey information to AgRP or POMC neurons, they were infected with PRVB177. This virus travels retrogradely across multiple synapses in a time-dependent manner after infecting Cre-expressing neurons.

These studies show that both AgRP and POMC neurons receive information indirectly from the OC. The OC contains numerous anatomically distinct areas. Neurons upstream of AgRP or POMC neurons were seen in some of those areas, but not others, suggesting the potential involvement of only some OC areas in the control of appetite.

The results further indicate that different OC areas communicate with AgRP versus POMC neurons. While one OC area sends information to both, four other OC areas send signals only to AgRP or POMC neurons. Thus, AgRP and POMC neurons are likely to receive different sets of olfactory information.

The respective functions of different OC areas are largely obscure (15, 16). However, one (AmPir) is linked to physiological fear responses (22) and another (PLCo) to innate attraction and aversion (41). It is conceivable that OC areas upstream of AgRP or POMC neurons similarly have distinct effects on appetite, particularly the PLCo, which we saw indirectly upstream of AgRP but not POMC neurons. These considerations emphasize the importance of further exploration to elucidate the roles of different OC areas upstream of AgRP and POMC neurons in appetite regulation.

These analyses extended the main olfactory pathway to consider inputs from the VA, which processes olfactory sensory signals about social cues from the VNO (15, 16, 23). Upstream neurons in the two areas of the VA suggest these areas as influencers of both AgRP and POMC neurons. It also opens the possibility that social cues detected by the VNO could have an impact on appetite and energy metabolism, integrating social stimuli with fundamental physiological responses.

These studies show that AgRP and POMC neurons receive more direct input from numerous nonolfactory brain areas, primarily in the hypothalamus. These neurons have the potential to relay information from the OC to the appetite neurons. Most or all projection neurons in the OC are excitatory glutamatergic neurons (22). However, they could activate either excitatory or inhibitory neurons presynaptic to AgRP or POMC neurons and thereby cause either activation or suppression of the appetite neurons.

In exploring the molecular basis of these connections, we found a rich diversity of GPCRs in AgRP neurons. These receptors, which include those for neurotransmitters like glutamate and GABA, as well as for various neuropeptides, illustrate the complexity of input integration at the neuronal level. The differential expression of these GPCRs among AgRP neurons suggests a mechanism where individual AgRP neurons may be selectively responsive to a limited spectrum of those signaling molecules, potentially directing varied physiological responses. AgRP neurons also express ionotropic (“fast neurotransmitter”) receptors for glutamate and GABA, the major excitatory and inhibitory neurotransmitters in the brain, respectively. The GPCRs could play a role in enhancing or dampening these rapid responses to fine-tune the cell’s responses to multiple signals.

The importance of the AgRP neuron GPCRs and GPCR ligands revealed in these experiments is supported by genetic studies that link these molecules to obesity and energy homeostasis in humans or mice (42). These include human obesity-associated polymorphisms in three receptors: CRHR1 (43), MCHR1 (44–46), and MC3R (47–50). In mice, they include three receptors whose deletion increases body weight and adiposity [Mc3r (51, 52), Npy1r (53), and Npy5r (54)] and two that have the opposite effect [Ghsr (55, 56) and Cnr1 (57)]. The ligands of identified receptors can also have effects when modulated, with overexpression of Crh increasing body weight (58) and deletion of Adcyap1 decreasing it (59). These associations between AgRP neuron receptors and their ligands and genes that alter energy homeostasis emphasize the potential importance of these molecules as pharmaceutical targets.

By defining the specific ligands of AgRP neuron receptors and the distinct subsets of upstream neurons that express these ligands, these studies uncovered molecular genetic tools to further dissect the roles of these neurons in controlling appetite. This foundational knowledge sets the stage for future studies aimed at understanding how different neural circuits integrate multiple forms of input, including olfactory signals, to regulate appetite. It also provides a rich complement of receptors and ligands that offer potential therapeutic targets for disorders of appetite and energy homeostasis.

## Materials and Methods

### Animals.

C57BL/6J wild type, AgRP-IRES-Cre (34) (Jax stock no: 012899), Ai6 (35) (Jax stock#: 007906), Pomc-eGFP (60) (Jax stock#: 009593), and POMC-Cre (61) (Jax stock#: 005965) mice were purchased from the Jackson Laboratory. All procedures involving mouse handling were approved by the Fred Hutchinson Cancer Center Institutional Animal Care and Use Committee.

### Viral Vectors.

PRVB177 was constructed as previously described (22). Briefly, a CMV promoter, a flexstop-flanked sequence encoding a PRV thymidine kinase (TK) fused with a hemagglutinin (HA) epitope tag, and an SV40 polyadenylation signal, were cloned into PRV TK-BaBlu, a TK-deleted PRV Bartha strain between its gG locus sequences matching 5′ and 3′ to the lacZ sequence. Recombinant virus clones were selected and confirmed as described previously (22). Recombinant PRVs were propagated in PK15 cells (ATCC) using a multiplicity of infection (M.O.I.) = 0.1 ~ 0.01. Three days after infection, cells were harvested by scraping. Material was frozen using liquid nitrogen and then quickly thawed in a 37 °C water bath 3 times. Cell debris was then removed by centrifugation twice at 1,000 × g for 5 min. The titer of supernatant was determined using standard plaque assays on PK15 cells, with titers expressed in plaque-forming units (PFU).

### Stereotaxic Injection.

Stereotaxic injection was performed as previously described (22). Two μL of virus suspension (1 to 2 × 109 PFU) was injected into the brains of mice aged 2 to 6 mo at 100 nL per minute. Targeted brain areas were referenced based on a stereotaxic atlas (62) using a Stereotaxic Alignment System (David Kopf Instruments). Mice were treated with an inhalation anesthesia of 2.5% Isoflurane during injection. Animals were singly housed with regular 12 h dark/light cycles in the presence of food and water ad libitum after recovery.

### Staining for Neuropeptide mRNA and PRVB177 (HA).

Experiments were performed as described previously (22). For perfusion, 4% paraformaldehyde (PFA) was used to perfuse animals transcardially. Brains were dissected out and then soaked in 4% PFA overnight. After soaking in 30% sucrose for 48 h, the brains were frozen in OCT (Sakura) and stored at −80 °C before sectioning. For fresh frozen tissues, animals were decapitated immediately after cervical dislocation. Fresh brains were dissected out, snap frozen in isopentane mixed with dry ice, and kept at −80 °C. Brains were sectioned into 20 μm coronal sections using a cryostat.

For detection of PRV-positive neurons, brain sections were incubated with biotinylated mouse anti-HA antibodies (BioLegend, #901505, 1:300) at 4 °C overnight or at 37 °C for an hour. Sections were then incubated with 0.5 μg/mL DAPI (Sigma) and Alexa488 Streptavidin (Thermo Fisher, 1:1,000) at room temperature for 1 h followed by coverslipping with Fluoromount-G (Southern Biotech). For double staining of PRV-infected neurons expressing neuropeptides, coding regions of neuropeptide genes were amplified from mouse brain cDNA using PCR and cloned into the pCR4 TOPO vector (Thermo Fisher). Digoxigenin (DIG)-labeled cRNA probes (riboprobes) were prepared using the DIG RNA Labeling Mix (Roche). Twenty μm coronal cryostat sections of mouse brains frozen in OCT were hybridized to DIG-labeled cRNA probes at 56 °C for 13 to 16 h. After hybridization, brain sections were washed in 5 × SSC and 0.2 × SSC at 63 °C for 30 min each consecutively. Sections were incubated with POD-conjugated anti-DIG antibodies (Roche, #11207733910, 1:2,000) and biotinylated anti-HA antibodies (BioLegend, #901505, 1:300) at 4 °C for overnight or at 37 °C for an hour and treated using the TSA-plus Cy3 kit (Perkin Elmer) according to the manufacturer’s instructions. Sections were then incubated with 0.5 μg/mL DAPI and Alexa488 Streptavidin (Thermo Fisher, 1:1,000) at room temperature for 1 h. Slides were coverslipped with Fluoromount-G.

### scRNA-seq.

AgRP-IRES-Cre mice (34) were crossed with Ai6 (35) mice, which have Cre-dependent expression of eGFP, to generate AgRP-Cre:Ai6 mice. Arcuate nuclei of AgRP-Cre:Ai6 mice were then dissected out and dissociated into single cells as previously described (37). Dissociated single cells were placed on a coverslip and visualized under a fluorescent microscope and GFP-labeled single cells were manually isolated using a micropipette and each one transferred into a tube. Single cells were lysed, mRNAs reverse transcribed, and cDNA libraries for sequencing were generated using the Illumina TruSeq DNA Sample Prep kit similar to our published protocols (27, 36, 37). A total of 18 cells with good quality cDNAs were analyzed by PCR to confirm the expression of AgRP and NPY. The following intron-spanning primers were used: AgRP-5’, CAACTGCAGACCGAGCAGAAG, AgRP-3’-GCAGCAAGGTACCTGCTGTC, NPY-5’, GGCACCCAGAGCAGAGCAC, and NPY-3’, CCAGAATGCCCAAACACACG. cDNAs that confirmed expression of markers were processed for Illumina deep sequencing using an Illumina Hiseq 2500 instrument, similar to our previously published methods (37). Sequenced reads were mapped to the mouse genome (mm10) to generate Fragments Per Kilobase of exon per Million mapped fragments (FPKM) using standard methods (Tophat and Cufflinks) (63, 64). Cells were sequenced at an average depth of 9,219,872 reads per cell. An average of 3,952 genes per cell were detected at ≥1FPKM.

We then generated a list of receptor genes in 18 individual AgRP+ neurons with FPKM ≥1, which was used to construct an expression matrix. Lists of ligand-gated ion channels and GPCRs for neurotransmitters or neuromodulators were obtained from the International Union of Basic and Clinical Pharmacology/British Pharmacological Society website (www.guidetopharmacology.org).

To analyze coexpression of GPCRs, pairwise gene–gene coexpression frequencies were computed across the 18 AgRP+ neurons. The resulting coexpression matrix was plotted using Matplotlib v3.8. All data visualization and downstream analyses were performed in Python.

To analyze data from a larger number of AgRP+ neurons, we used publicly available single-cell transcriptome data from the Allen Brain Cell Atlas (41) (https://brain-map.org/bkp/explore/abc-atlas), which provides an imputed MERFISH dataset covering the adult C57BL/6J mouse brain. The imputed, cell-by-gene expression matrix (downloaded from the “ABC Atlas Cache”) was processed using Scanpy v1.9 and AnnData v0.10 in Python 3.12. AgRP+ cells (4,174 cells) annotated to the arcuate nucleus (ARH) (ARC) were extracted from the full dataset, and expression values for the selected genes were log-normalized and z-scored per cell, using a threshold of ≥1 CPM (counts per million). Expression was analyzed for the GPCRs identified in our smaller dataset and the percentage of neurons expressing those GPCRs in the two datasets were compared. An expression matrix was constructed for the shared GPCRs in the Allen dataset using 50 randomly chosen AgRP+ neurons from the set of 4,174 cells.

To analyze coexpression of GPCRs in the Allen dataset, those 50 AgRP+ cells were processed in the same manner as in the smaller dataset. The resulting coexpression matrix was plotted using Matplotlib v3.8, as in the smaller dataset, with data visualization and downstream analyses performed in Python.

### Cell Counting.

Stained brain sections were imaged with an AxioImager.Z1 microscope. Brain structures were identified based on a mouse brain atlas (62). Numbers of PRV-infected cells in brain areas of every fifth section were counted. To acquire approximate total number of cells in each brain area of an animal, brain areas were judged to contain upstream neurons if they contained ≥2 labeled neurons in ≥50% of animals. All data are shown as the mean ± SEM. Neuropeptide counts were confirmed with TissueFax in Fred Hutch Cancer Center Shared Resources.

### Abbreviations for Brain Areas.

Modified based on Franklin and Paxinos (62).

Acb: accumbens nucleus
ACo: anterior cortical amygdaloid area
AH: anterior hypothalamic area
AHi: amygdalohippocampal area
AmPir: amygdalo-piriform transition area
AON: anterior olfactory nucleus
aPir: piriform cortex, anterior part
ARC: arcuate hypothalamic nucleus
CA1: field CA1 of the hippocampus
CEnt: caudomedial entorhinal cortex
Coxe: cortex-amygdala transition zone
DA: dorsal hypothalamic area
DG: dentate gyrus
DIEnt: dorsal intermediate entorhinal cortex
DMH: dorsomedial hypothalamic nucleus
DTM: dorsal tuberomammillary nucleus
LEnt: lateral entorhinal cortex
LH: lateral hypothalamic area
LPO: lateral preoptic area
ME: median eminence
MEA: medial amygdala
MEnt: medial entorhinal cortex
MM: medial mammillary nucleus, medial part
MPA: medial preoptic area
MPO: medial preoptic nucleus
MTu: medial tuberal nucleus
NLOT: nucleus of the lateral olfactory tract
OC: olfactory cortex
OT: olfactory tubercle
Pe: periventricular hypothalamic nucleus
PeF: perifornical nucleus
PH: posterior hypothalamic nucleus
PLCo: posterolateral cortical amygdaloid area
PLH: peduncular part of lateral hypothalamus
PMCo: posteromedial cortical amygdala
PMD: premammillary nucleus, dorsal part
PMV: premammillary nucleus, ventral part
pPir: piriform cortex, posterior part
PSTh: posterior subthalamic nucleus
PVN: paraventricular nucleus of the hypothalamus
RCH: retrochiasmatic area
RM: retromammillary nucleus
SCh: suprachiasmatic nucleus
SHy: septohypothalamic nucleus
SO: supraoptic nucleus
StHy: striohypothalamic nucleus
Te: terete hypothalamic nucleus
TT: tenia tecta
TuLH: tuberal region of lateral hypothalamus
VA: vomeronasal amygdala
VEn: ventral endopiriform claustrum
VIEnt: ventral intermediate entorhinal cortex
VMH: ventromedial hypothalamic nucleus
VTM: ventral tuberomammillary nucleus

## Supplementary Material

Appendix 01 (PDF)

## Data Availability

Raw sequencing data related to this study are archived in the National Center for Biotechnology Gene Expression Omnibus (GEO) database prior to publication (GEO accession number: GSE227909) (65).
