# Supplementary material for: Olfactory inputs to appetite neurons in the hypothalamus
Source: Proc Natl Acad Sci U S A. 2026 Jan 27;123(5):e2524926123. doi: 10.1073/pnas.2524926123 (PMC12867749; doi:10.1073/pnas.2524926123)
Supplement: Supplementary file 1 — Appendix 01 (PDF) [file pnas.2524926123.sapp.pdf]

PNAS

Supporting Information for

Olfactory inputs to appetite neurons in the hypothalamus

Donghui Kuang[1], Naresh K. Hanchate[1,3], Chia-Ying Lee[1,4], Ashley Heck[1,4],

Xiaolan Ye[1], Michidsaran Erdenebileg[1,5], Charu Mehta[1], Md Mehedi Hassan[1],

Manu Setty[1], and Linda B. Buck[1,2]

Correspondence and requests for materials should be addressed to L.B.B.

(Email: lbuck@fredhutch.org, phone : 206-667-6316)

This PDF file includes:

Figures S1 to S8

Figures

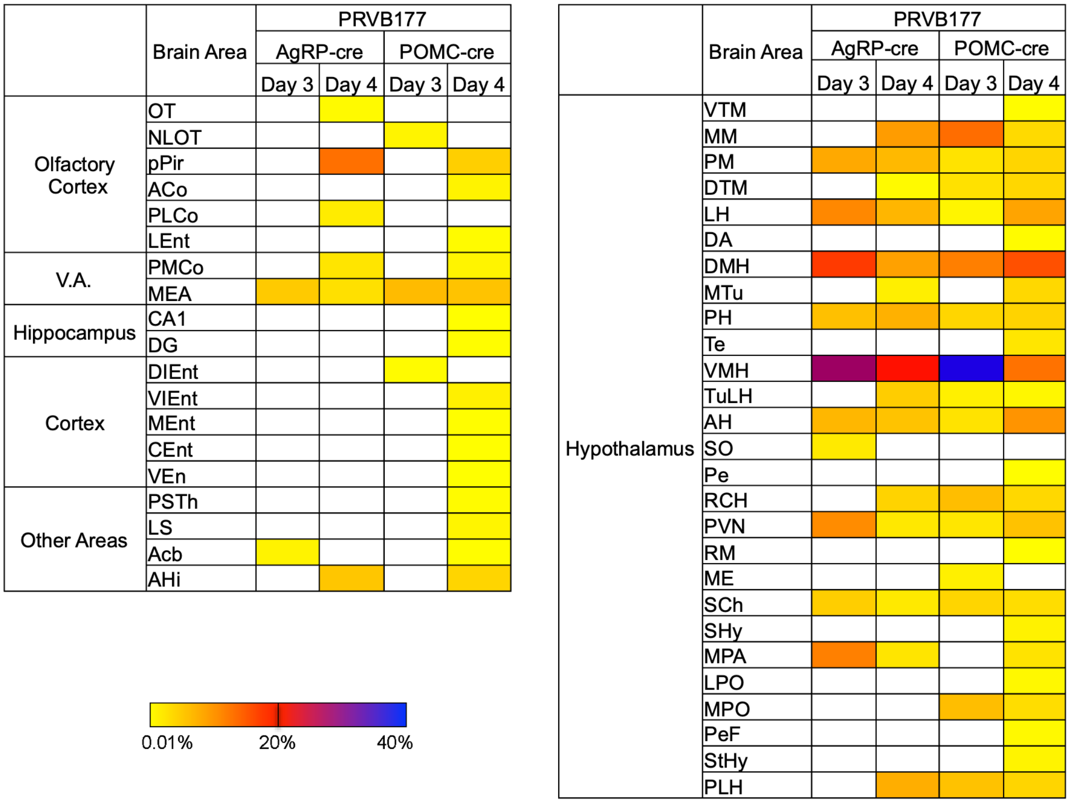

Fig. S1. Heat map of brain areas with neurons upstream of AgRP or POMC neurons. Colored boxes indicate approximate percentages of all non-ARC PRV+ neurons in individual brain areas, denoted by color intensity (white indicates none) on day 3 or 4 after ARC injection of AgRP-Cre or POMC-Cre mice with PRVB177. Sample sizes as in Fig. 2. Areas with PRV+ neurons on day 3 are more directly upstream of AgRP or POMC neurons. Areas with PRV+ neurons on day 4 but not day 3 are indirectly upstream of AgRP or POMC neurons. See Methods for full names of abbreviated brain areas.

AgRP-Cre, Day 3, PRV+

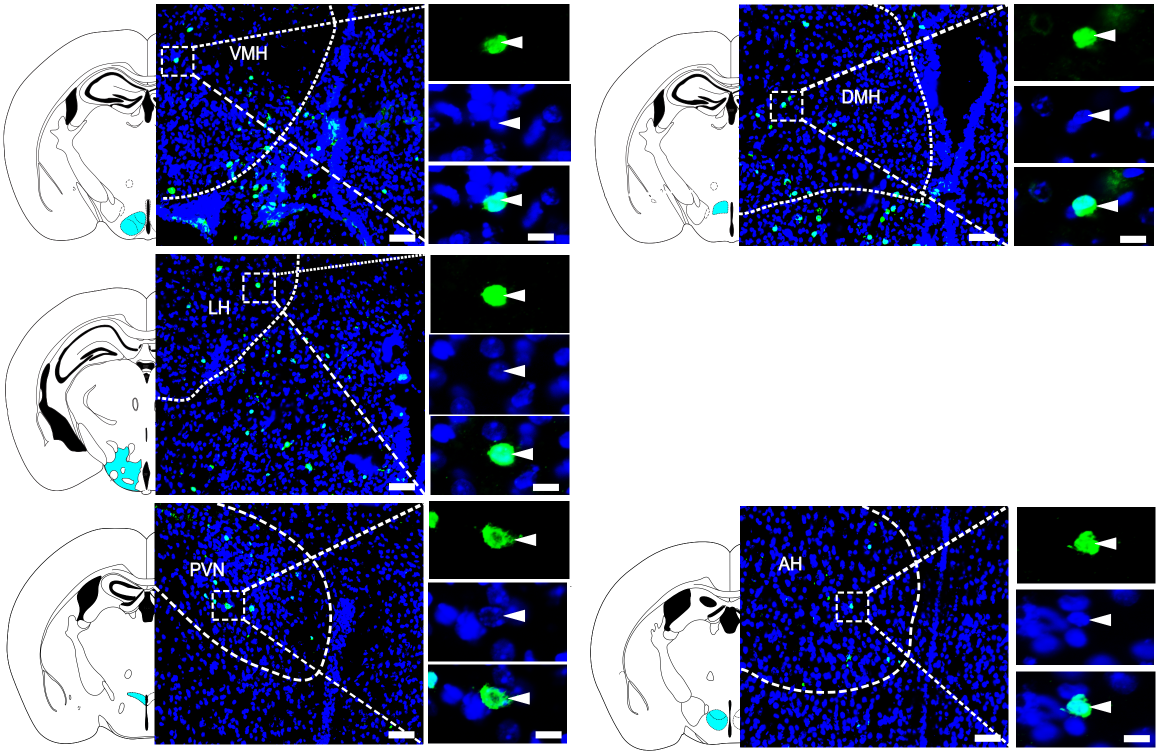

Fig. S2. Non-olfactory areas with PRV+ neurons more directly upstream of AgRP neurons. Images of cells immunostained for PRV (HA) (green) in different non-olfactory areas on day 3 post-injection of AgRP-Cre mice. DAPI counterstain, blue. Corresponding areas on diagrams are labeled with cyan. Dotted lines indicate locations of brain areas. Boxed areas are shown at higher magnification at right (top, PRV; middle, DAPI; bottom, merged). Scale bars, 100 μm (middle) and 20 μm (right).

POMC-Cre, Day 3, PRV+

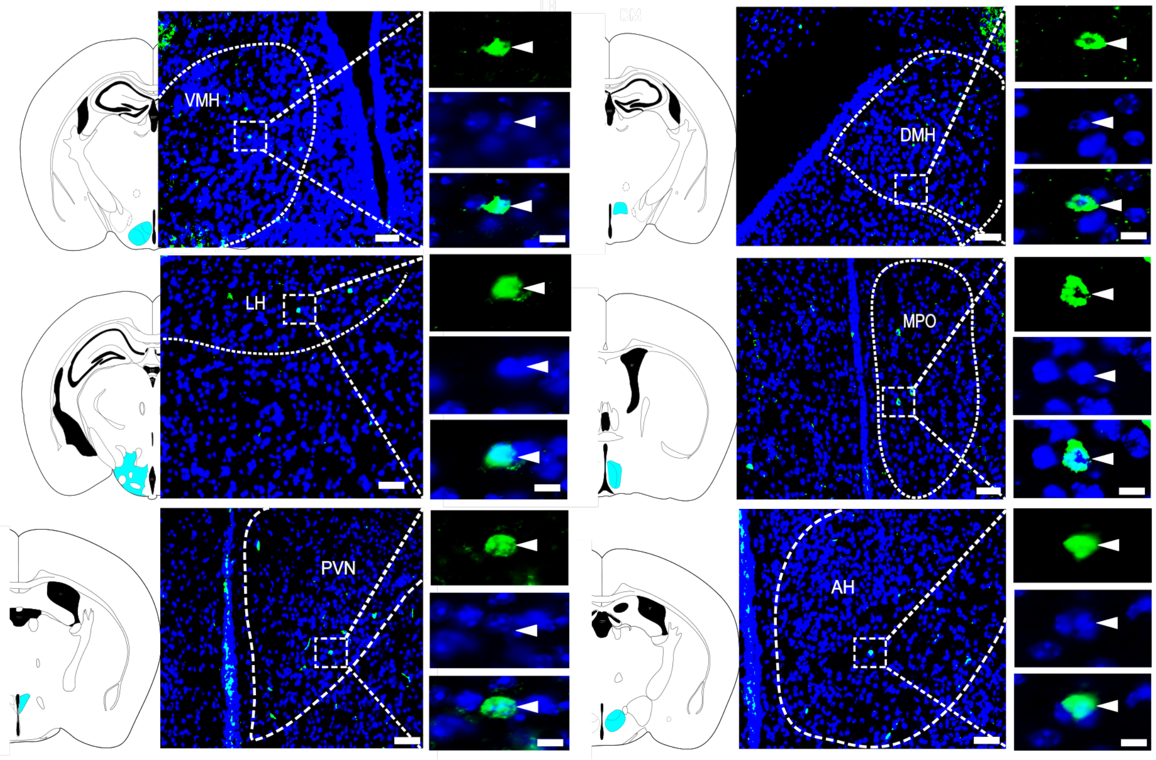

Fig. S3. Non-olfactory areas with PRV+ neurons more directly upstream of POMC neurons. Images of cells immunostained for PRV (HA) (green) in different non-olfactory areas on day 3 post-injection of POMC-Cre mice. DAPI counterstain, blue. Corresponding areas on diagrams are labeled with cyan. Dotted lines indicate locations of brain areas. Boxed areas are shown at higher magnification at right (top, PRV; middle, DAPI; bottom, merged). Scale bars, 100 μm (middle) and 20 μm (right).

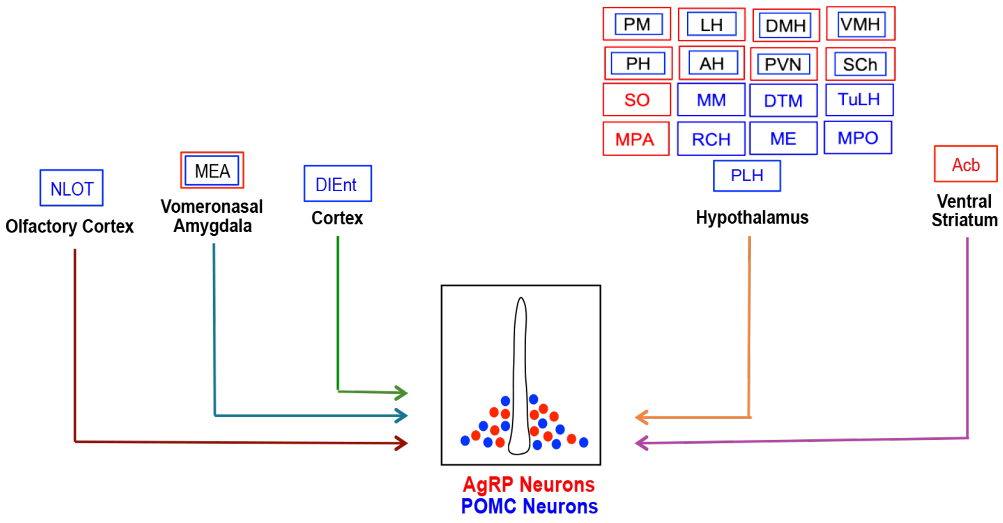

Fig. S4. Anatomical map of more direct inputs to AgRP and POMC neurons. Schematic shows brain areas containing neurons more directly upstream of AgRP or POMC neurons, illustrated in red and blue, respectively. Twelve areas (including 11 non-olfactory) contain neurons upstream of AgRP neurons while eighteen (including 16 non-olfactory) areas contain neurons upstream of POMC neurons. Nine areas (including 8 non-olfactory) contain neurons upstream of both AgRP and POMC neurons. Olfactory areas: NLOT, MEA.

Ligand	Gene	% of Neurons
GABA	*Gabbr1*	83
Pacap, Vip	*Adcyap1r1*	78
neuropeptide Y	*Npy2r*	78
calcitonin, Cgrp, amylin, adrenomedullin	*Calcr*	72
opiates	*Oprl1*	61
histamine	*Hrh3*	56
glutamate	*Grm7*	44
melonocortin	*Mc3r*	39
neuronostatin	*Gpr107*	28
serotonin	*Htr1a*	28
ghrelin	*Ghsr*	28
GABA	*Gabbr2*	22
prokineticin	*Prokr2*	22
cortistatin, somatostatin	*Sstr1*	22
melanin-concentrating hormone	*Mchr1*	17
epinephrine, norepinephrine	*Adra1a*	17
neuropeptide Y	*Npy1r*	17
Chemokine	*Cxcr6*	11
Corticotropin-releasing factor	*Crhr1*	11
Orexin	*Hcrtr1*	11
ADP/ATP	*P2ry14*	11
ADP/ATP	*P2ry1*	11
glutamate	*Grm5*	11
thrombin	*F2r*	11
cannabinoid	*Cnr1*	11
Galanin	*Galr2*	6
serotonin	*Htr1b*	6
cortistatin, somatostatin	*Sstr4*	6
epinephrine, norepinephrine	*Adra2a*	6
neuropeptide FF	*Npffr1*	6
neuropeptide FF	*Npffr2*	6
prostanoid	*Ptger1*	6
neuropeptide Y	*Npy5r*	6
opiates	*Oprk1*	6
lysophospholipid	*S1pr3*	6
serotonin	*Htr6*	6
neurotensin	*Ntsr2*	6
lysophosphatidic acid	*Lpar1*	6
Bradykinin	*Bdkrb2*	6

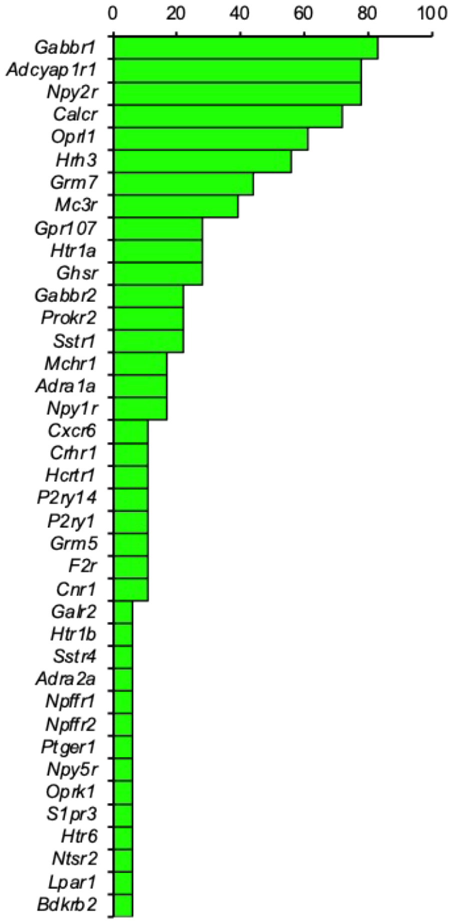

Fig. S5. GPCR expression in scRNA-seq data. Chart displays the percentages of AgRP neurons in the scRNA-seq dataset that express genes encoding GPCRs with known ligands, as indicated. The percentages are visually represented as green bars on the right.

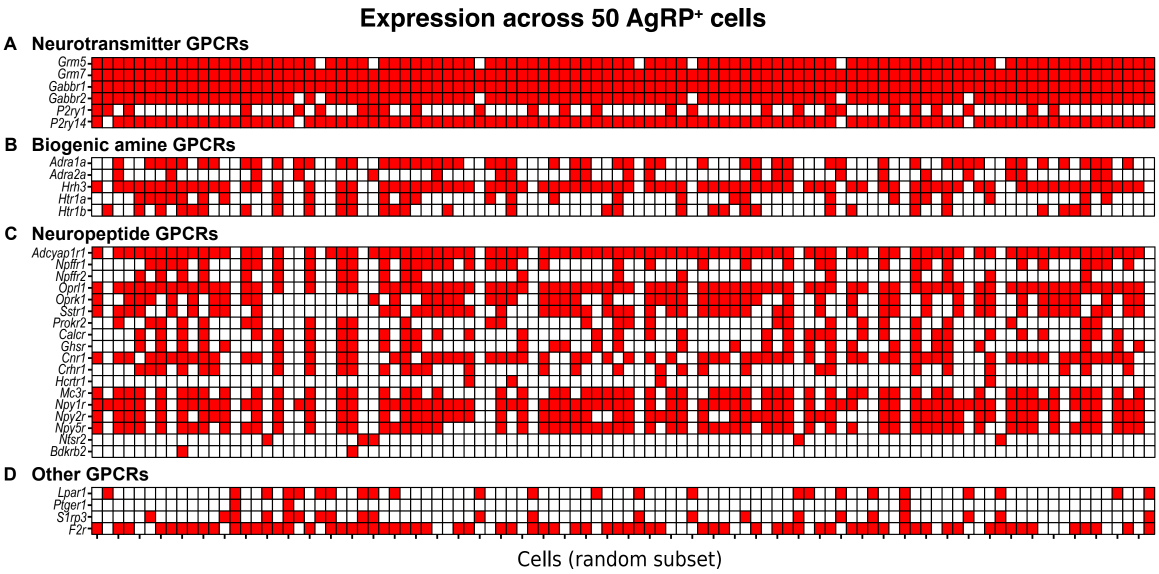

Fig. S6. GPCR expression in AgRP neurons in the Allen Brain Cell Atlas. An expression matrix shows transcriptome data for 50 randomly selected AgRP+ neurons of 4174 found in the arcuate nucleus in the Allen Brain Cell Atlas. Data are shown for 39 GPCRs found in AgRP neurons in both this dataset and a smaller dataset from manually isolated cells. They include GPCRs for neurotransmitters (A), biogenic amines (B), neuropeptides (C), and other ligands (D). Red boxes indicate expression in individual AgRP neurons of receptor genes indicated on the Y-axis. Single neurons express multiple receptors, and those receptors are expressed in different combinations in different neurons.

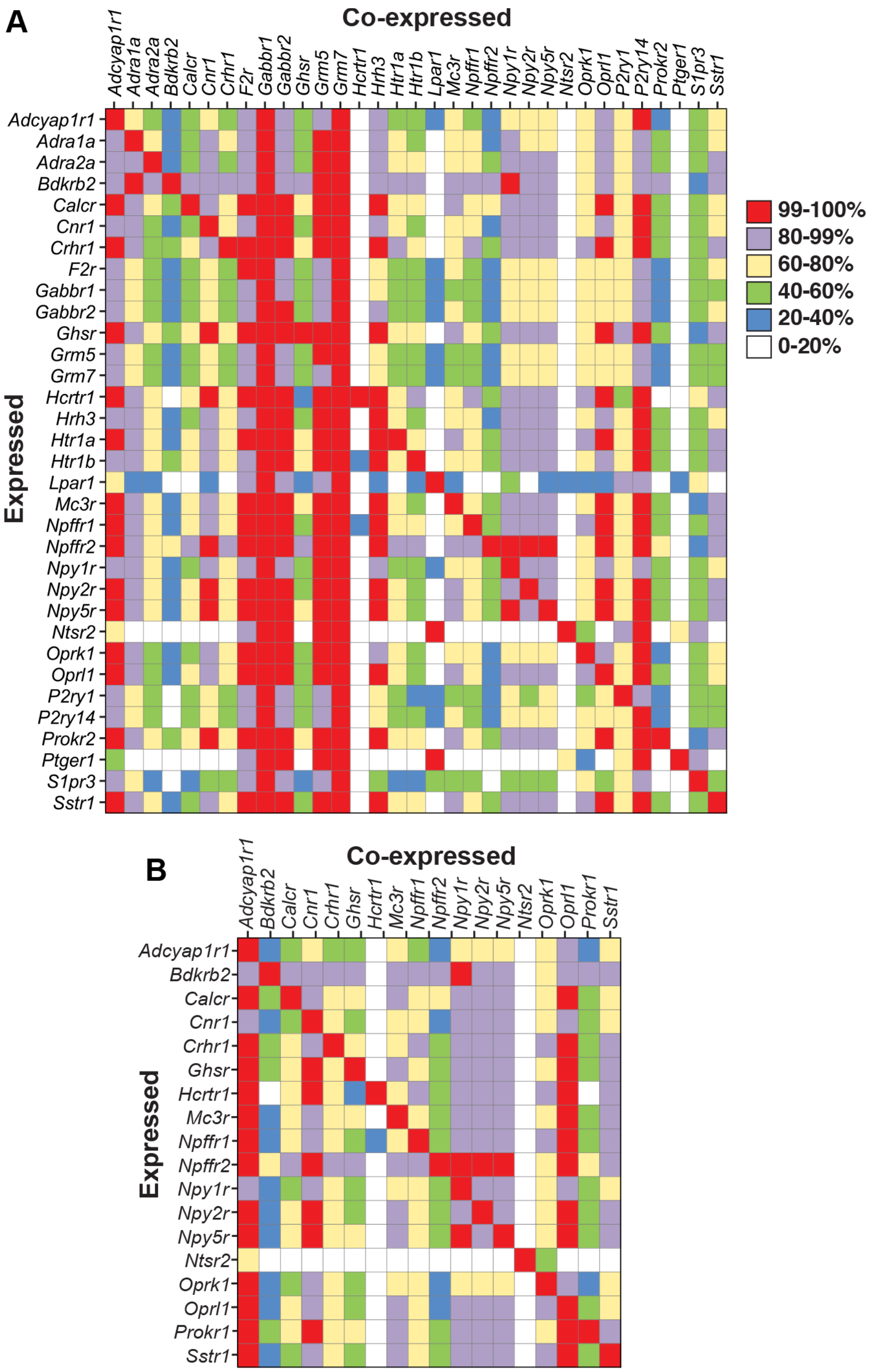

Fig. S7. Coexpression of GPCRs in AgRP neurons. Coexpression data are shown for the 50 AgRP neurons in Fig. S6. Coexpression is shown for all GPCRs (A) or only neuropeptide GPCRs (B). Expressed GPCR genes are indicated on the y-axis and

coexpressed GPCR genes on the x-axis. Box colors indicate percentages of neurons expressing different genes (y-axis) that coexpress genes on the x-axis.

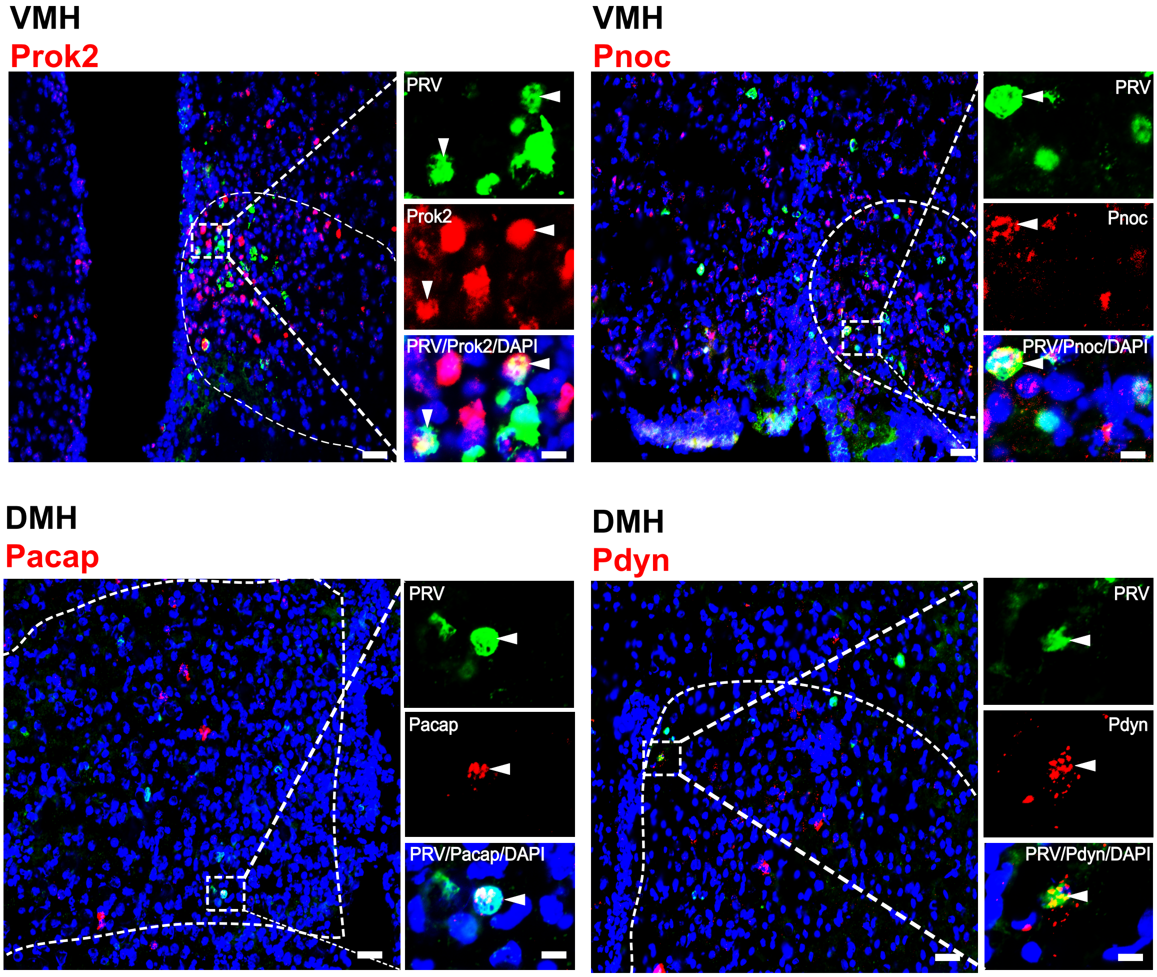

Fig. S8. Expression of individual neuropeptides in single PRV+ cells in AgRP-Cre mice. Photographs show expression of individual neuropeptides (Prok2, Pnoc, Pacap, or Pdyn) in single PRV+ neurons in VMH or DMH. PRV+, green; neuropeptide+, red; DAPI+, blue. Brain areas are indicated by dotted lines. Boxed areas are shown at higher magnification at right. Scale bars, 100 μm (left) and 20 μm (right).
